# Confirmatory structural validation and refinement of the Recurrent Urinary Tract Infection Symptom Scale

**DOI:** 10.1002/bco2.297

**Published:** 2023-10-04

**Authors:** Abigail F. Newlands, Melissa Kramer, Lindsey Roberts, Kayleigh Maxwell, Jessica L. Price, Katherine A. Finlay

**Affiliations:** ^1^ School of Psychology and Clinical Language Sciences University of Reading Reading UK; ^2^ Live UTI Free Ltd Dublin Ireland; ^3^ School of Psychology University of Buckingham Buckingham UK; ^4^ Department of Psychology, Faculty of Natural Sciences University of Stirling Stirling UK

**Keywords:** bifactor model, chronic pain, item response theory, patient‐reported outcomes, recurrent urinary tract infection, women's health

## Abstract

**Objectives:**

To confirm the structural validity of the Recurrent Urinary Tract Infection Symptom Scale (RUTISS), determining whether a bifactor model appropriately fits the questionnaire's structure and identifying areas for refinement. Used in conjunction with established clinical testing methods, this patient‐reported outcome measure addresses the urgent need to validate the patient perspective.

**Patients and methods:**

A clinically and demographically diverse sample of 389 people experiencing recurrent UTI across 37 countries (96.9% female biological sex, aged 18–87 years) completed the RUTISS online. A bifactor graded response model was fitted to the data, identifying potential items for deletion if they indicated significant differential item functioning (DIF) based on sociodemographic characteristics, contributed to local item dependence or demonstrated poor fit or discrimination capability.

**Results:**

The final RUTISS comprised a 3‐item symptom frequency section, a 1‐item global rating of change scale and an 11‐item general ‘rUTI symptom and pain severity’ subscale with four sub‐factor domains measuring ‘urinary symptoms’, ‘urinary presentation’, ‘UTI pain and discomfort’ and ‘bodily sensations’. The bifactor model fit indices were excellent (root mean square error of approximation [RMSEA] = 0.041, comparative fit index [CFI] = 0.995, standardised root mean square residual [SRMSR] = 0.047), and the mean‐square fit statistics indicated that all items were productive for measurement (mean square fit indices [MNSQ] = 0.64 – 1.29). Eighty‐one per cent of the common model variance was accounted for by the general factor and sub‐factors collectively, and all factor loadings were greater than 0.30 and communalities greater than 0.60. Items indicated high discrimination capability (slope parameters > 1.35).

**Conclusion:**

The 15‐item RUTISS is a patient‐generated, psychometrically robust questionnaire that dynamically assesses the patient experience of recurrent UTI symptoms and pain. This brief tool offers the unique opportunity to enhance patient‐centred care by supporting shared decision‐making and patient monitoring.

## 1 INTRODUCTION

Urinary tract infections (UTIs) affect more than 400 million people worldwide each year,^1^ with between 24% and 50% of females experiencing a recurrence within 1 year.^2^ Defined by the European Association of Urology as experiencing at least two UTIs in 6 months or at least three in a year,^3^ recurrent UTI (rUTI) is associated with significant symptom burden and reduced quality of life.^4^, ^5^ rUTI‐specific patient‐reported outcome measures (PROMs) are required to validate the unique patient experience of rUTI symptoms. Microbiological research indicates that current clinical approaches, such as standard urine culture, may present results discrepant with symptoms.^6^ PROMs would allow clinicians and researchers to consider the patient perspective in conjunction with evaluation of clinical outcomes, thus addressing the urgent need to improve patient monitoring and rUTI management.^7^, ^8^

**FIGURE 1:**
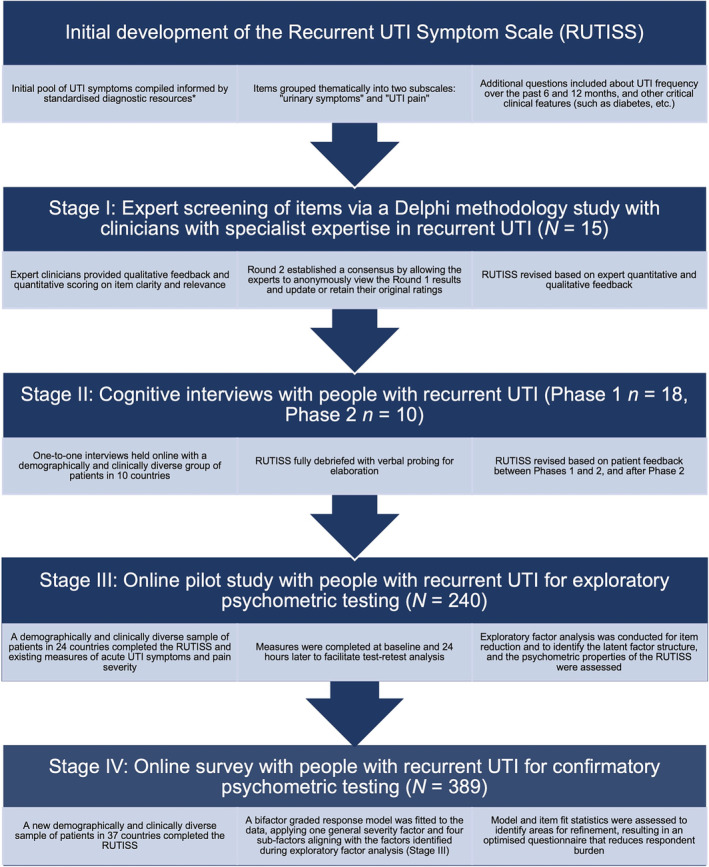
Methodology used to develop and validate the Recurrent Urinary Tract Infection Symptom Scale (RUTISS). The current study reports the methodology and results from Stage IV. Results from Stages I–III are published in Newlands et al.^9^ *Diagnostic resources including the NHS and National Institute for Health and Care Excellence (NICE) guidelines on UTIs.^4^

The Recurrent UTI Symptom Scale (RUTISS) is a novel PROM exploring frequency of UTI symptoms, severity of rUTI symptoms and pain, and patient‐perceived improvement in symptoms (via application of a global rating of change, GRC, scale).^9^ The RUTISS was developed and pilot‐tested with extensive input from heterogeneous, international patient and expert clinician samples, robustly following gold‐standard PROM development recommendations by the COnsensus‐based Standards for the selection of health Measurement INstruments (COSMIN) initiative (see Figure 1 for development methodology; Stages I–III have been published in Newlands et al.).^9^, ^10^, ^11^ Exploratory factor analysis (EFA) of initial pilot data resulted in a four‐factor structure comprising ‘urinary symptoms’, ‘urinary presentation’, ‘UTI pain and discomfort’ and ‘bodily sensations’.^9^ The RUTISS demonstrated excellent test–retest reliability (intraclass correlation coefficient, ICC > 0.73), strong concurrent validity with related urinary symptom and pain measures (Spearman's ρ > 0.60), excellent internal consistency (Cronbach's α > 0.87) and excellent content validity (content validity indices for items, I‐CVI > 0.75),^9^ meeting recommendations by COSMIN.^10^, ^11^

A bifactor model hypothesises that (i) there is a general factor that explains the shared variance between all the items, and (ii) there are two or more specific, uncorrelated factors that each account for the unique influence of the specific construct over and above the general factor.^12^, ^13^ Bifactor modelling enables the separate evaluation of variance explained by a common, general factor and specific factors.^13^ A well‐fitting bifactor structure therefore allows questionnaire administrators to compute general scores based on all the items represented by a general latent trait, as well as individual domain scores for items represented by specific traits.^12^ Thus, the current study aimed to build on preliminary testing of the RUTISS by confirming its structural validity, determining whether a bifactor model appropriately fits the questionnaire's structure and identifying areas for refinement.^12^ This confirmatory factor structure validation is the next step in the PROM development process and is a prerequisite for ongoing work to evaluate the questionnaire's clinical responsiveness and sensitivity to change.^10^ Under this model, it was hypothesised that the RUTISS would measure one general factor (‘rUTI symptom and pain severity’) and four sub‐factors based on the factors identified through EFA.^9^, ^12^

## 2 PATIENTS AND METHODS

### 2.1 Study design and participants

A cross‐sectional survey of adults with rUTI was conducted online, employing modern psychometric approaches including item response theory (IRT) to evaluate the factor structure of the RUTISS most effectively, making optimal refinements to minimise respondent burden (see Section 2.3).^12^, ^14^, ^15^ A total of 389 adults meeting the diagnostic criteria for rUTI (≥2 UTIs in 6 months or ≥3 UTIs in 12 months) completed the survey (96.9% female biological sex, *n =* 377; see Table 1 for demographic characteristics). Most participants (84.8%, *n =* 330) were recruited via newsletters, research notifications and social media posts from a key stakeholder group, Live UTI Free: a popular UTI patient advocacy and research organisation. The remainder were recruited via other UTI‐related online sources such as support groups (5.66%, *n =* 22) and other routes including participants sharing the study information on social media websites including Instagram, Facebook, Twitter and Reddit (9.51%, *n =* 37).

**TABLE 1:** Participant demographic characteristics.

Characteristic	*n*	%
Biological sex		
Female	377	96.9
Male	12	3.08
Gender		
Female	374	96.1
Male	12	3.08
Non‐binary	2	0.51
Prefer not to say	1	0.26
Country of residence		
United Kingdom	153	39.3
United States	147	37.8
Canada	26	6.68
Australia	8	2.06
Ireland	5	1.29
Greece	4	1.03
India	4	1.03
Spain	4	1.03
Other^a^	38	9.77
Ethnicity		
White (including Caucasian, White British, White European)	340	87.4
Asian (including Asian American, Asian British)	10	2.57
Hispanic or Latino American	5	1.29
Mixed ethnicity or multiple ethnic groups	4	1.03
Black (including African, African American, Caribbean, Black British)	3	0.77
Native Hawaiian or other Pacific Islander	2	0.51
Other ethnicity	4	1.03
Prefer not to say	21	5.40
Fluency in English		
Native or bilingual	337	86.6
Advanced or proficient	52	13.4
Relationship status		
Married or in a civil partnership	199	51.2
In a relationship (unmarried)	117	30.1
Single	44	11.3
Divorced	14	3.60
Widowed	6	1.54
Separated	5	1.29
Other	2	0.51
Prefer not to say	2	0.51
Highest level of education		
Some high school/secondary school	7	1.80
High school/secondary school	65	16.7
Bachelor's degree or equivalent	168	43.2
Master's degree or equivalent	104	26.7
Doctoral level training or equivalent	16	4.11
Other professional qualification(s)	22	5.66
Prefer not to say	7	1.80
Annual household income (GBP)		
No current income	12	3.08
£1–£9999	15	3.86
£10 000–£24 999	37	9.51
£25 000–£49 999	102	26.2
£50 000–£74 999	48	12.4
£75 000–£99 999	40	10.3
£100 000 or more	59	15.2
Prefer not to say	76	19.5

*Note*: *N* = 389.

^a^ Other countries where *n* ≤ 3 comprise the following 29 countries listed alphabetically: Angola, Argentina, Austria, The Bahamas, Belgium, Croatia, Czech Republic, Denmark, Finland, France, Germany, Iceland, Israel, Italy, Jersey, Malawi, Mexico, Netherlands, New Zealand, Nigeria, Norway, Romania, Serbia, Slovakia, South Africa, Sweden, Thailand, Turkey, and Ukraine.

Participants were excluded if they did not meet the rUTI diagnostic criteria, reported a current diagnosis of interstitial cystitis, or did not have native or advanced fluency in the English language. A minimum sample size of 250 was sought to facilitate multidimensional IRT analysis with a graded response model (GRM) for polytomous data.^16^ To help meet this goal, recruitment was supported by randomly selecting four participants out of the total sample (*N* = 389) to each receive one online shopping vouchers worth £25, in line with NIHR payment guidance for researchers.^17^, ^18^

Sampling adequacy was exceeded. Eighty‐three people only partially completed the survey, and 52 people were excluded for not meeting the eligibility criteria; thus, their data were not included in analysis (see Figure S1 for sampling flow diagram).

Participants were aged between 18 and 87 years old (*M* = 45.4, *SD* = 17.1), and mostly reported female biological sex (96.9%, *n =* 377; see Table 1). The sample resided in 37 countries, predominantly the United Kingdom (39.3%, *n* = 153) and the United States (37.8%, *n* = 147). Participants generally reported a high level of education, with almost three‐quarters having achieved a bachelor's degree or above (74.0%, *n* = 288). Financial status was generally high, with over a third of participants reporting an annual household income of £50 000 or more (37.9%, *n* = 147), beyond the United Kingdom's average of £31 400 in 2021.^19^ The mean number of UTI episodes reported in the last 6 months was 3.62 (*SD* = 2.90), and the mean in the past year was 7.06 (*SD* = 5.91).

### 2.2 Procedure

Participants reviewed an information sheet detailing the study aims and ethical issues before electronically providing their consent to take part (full ethical approval was received after review by the University of Reading School of Psychology and Clinical Language Sciences Research Ethics Committee, project reference no. 2022‐115‐KF). They then completed a demographics screening questionnaire, at which point ineligible participants were excluded from continuing the survey. Eligible participants proceeded to complete the preliminary RUTISS, a 28‐item self‐report questionnaire assessing frequency of UTI symptoms (3 items), patient‐perceived change in UTI symptoms (1 item: GRC scale), severity of urinary symptoms (7 items) and UTI pain (10 items) and status of critical clinical features such as diabetes and pregnancy (7 items).^9^ The GRC scale utilises an 11‐point scale ranging from −5 (‘very much worse’) to 0 (‘no change’) to +5 (‘very much better’). Severity of urinary symptoms and UTI pain is measured using an 11‐point Likert scale ranging from 0 (‘not present’) to 1 (‘very mild’) to 10 (‘extremely severe’). A debrief form was provided at the end of the survey to signpost participants to mental health support resources.

### 2.3 Data handling and statistical analysis

The dataset was screened for ineligible participants and missing data (see Figure S1), resulting in a final sample of 389 included datapoints for multidimensional IRT analysis. Full definitions and brief explanations of statistical terminology are provided in Table S1.

#### 2.3.1 Preliminary model identification

Multidimensional IRT analysis was conducted with the 17 items within the ‘urinary symptoms’ and ‘UTI pain’ subscales of the RUTISS.^9^ To determine whether the RUTISS measures general rUTI symptom and pain severity in addition to the four specific symptom‐based traits identified during EFA, a confirmatory bifactor model was specified. The proposed model therefore stipulated one general factor onto which all 17 items were expected to load and four orthogonal sub‐factors that aligned with the factors identified during EFA^9^: ‘urinary symptoms’ (items C1–C4), ‘urinary presentation’ (items C5–C7), ‘UTI pain and discomfort’ (items D1–D6) and ‘bodily sensations’ (items D7–D10). The data were fitted with a multidimensional GRM given the polytomous nature of the data based on an 11‐point Likert scale and its recommendation for use in the evaluation of PROMs.^16^, ^20^ Item parameters were estimated using the Metropolis‐Hastings Robbins‐Monro (MHRM) estimation method as the expected number of factors was greater than three.^12^ All IRT analyses were conducted in R using the *mirt* package.

#### 2.3.2 Model assumption checks

To assess the assumption of monotonicity and evaluate the use of the 11‐point Likert scale, the ordering of the intercept parameters (c) that govern the choice of the next category over the previous one (i.e., responding 10 vs. 9) was examined. To meet the assumption and demonstrate consistent use of the 11‐point scale, the intercept values were expected to successively decrease as the response categories, and therefore, the latent trait of severity increased.^12^ Intercept parameters are inversely proportional to threshold parameters (β), which are expected to successively increase alongside the response categories to satisfy the monotonicity assumption.^12^

The assumption of local independence expects all item responses to be uncorrelated after controlling for the latent trait. To assess this, Yen's *Q*_3_ statistics were computed for each pair of items within the bifactor model.^21^ *Q*_3_ statistics, which may be interpreted as residual correlation coefficients, greater than 0.30 may indicate local item dependence (LID) and therefore suggest violation of the assumption.^22^, ^23^ Item pairs with LID (*Q*_3_ > 0.30) suggest possible redundance or the existence of another shared dimension, which may result in small distortion of parameter estimates.^21^

Item invariance, or the absence of differential item functioning (DIF), was evaluated by conducting likelihood ratio Chi‐square analysis.^24^ Items were checked for performing differently within the model based on biological sex (female vs. male), age (older vs. younger than the median, 42 years old), household income (£25 000 or above vs. less than £25 000), level of education (university degree or above vs. school or lower) and current antibiotic use (yes vs. no).^24^ The *multipleGroup* and *DIF* functions within R's *mirt* package were applied, freely estimating the model parameters across each categorical group. The presence of DIF (or the absence of item invariance) was indicated by a statistically significant group difference (χ^2^, *p* < 0.05).^24^ The Bonferroni correction was utilised to adjust *p* values for the impact of multiple comparisons.^25^

#### 2.3.3 Model fit and performance

Standardised factor loadings for each item were expected to be greater than 0.30 and communalities greater than 0.60.^26^ Item fit was assessed by examining the mean‐square (MNSQ) outlier‐sensitive (outfit) statistics, with values falling between 0.50 and 2.00 indicating that they are acceptable for measurement.^27^ Item slope (or discrimination) parameters (α) were considered to determine which items performed best within the model in terms of differentiating between respondents' rUTI severity.^12^, ^28^ Higher slopes indicate stronger items with greater discrimination capability, expecting minimum α = 0.65 to suggest at least ‘moderate’ performance.^28^

The C_2_ statistic of goodness of fit for ordinal data was calculated, with a non‐statistically significant result indicating good model fit.^29^ Given that this test is sensitive to sample size, it is common to make model fit inferences based on the following indices: root mean square error of approximation (RMSEA_C2_; ‘good fit’ ≤ 0.06), comparative fit index (CFI; ‘good fit’ ≥ 0.95) and standardised root mean square residual (SRMSR; ‘good fit’ ≤ 0.06).^29^, ^30^

#### 2.3.4 Model refinement

The RUTISS was refined and finalised according to the following strategies. First, an item was proposed for deletion if it (i) indicated statistically significant DIF after correction for multiple comparisons (*p* < 0.05), (ii) demonstrated poor item fit with MNSQ outfit statistics less than 0.50 or greater than 2.00, (iii) demonstrated low discrimination capability with slope parameter (α) less than 0.65, (iv) did not load onto any factor with standardised loading > 0.30, or (v) obtained a communality (*h* ^2^) less than 0.60. Secondly, if LID was identified between a pair of items with *Q*_3_ > 0.30, one item from the pair was considered for deletion based on MNSQ fit statistics and standardised factor loadings. Thirdly, the RMSEA, CFI and SRMSR model fit indices were evaluated.

The bifactor GRM analysis was re‐run iteratively after making each proposed deletion, until a confirmed model was reached. The final, refined version of the RUTISS was created based upon this confirmatory bifactor model (see Table 2 for final included items; the full RUTISS and scoring instructions are available from the corresponding author).

**TABLE 2:** Final 15 items included in the Recurrent Urinary Tract Infection Symptom Scale (RUTISS).

Updated section/item number	Instruction/item
Section A: Symptom frequency	The following questions are about **how often you experience UTI symptoms** . Please consider UTIs that may or may not have been medically diagnosed.
A1	Have you had UTI symptoms that feel continuous and do not fully subside **for at least the past 3 months** ?
A2	Approximately how many **episodes** of UTI symptoms have you had in the **past 6 months**?
A3	Approximately how many **episodes** of UTI symptoms have you had in the **past 12 months**?
Section B: Global rating of change	The following questions are about any **change** in your UTI symptoms.
B1	Please consider how you typically experience UTI symptoms. To what extent have your UTI symptoms over the PAST 24 HOURS been **better or worse than your typical experience** ?
Section C: Urinary symptoms and pain or discomfort	The following questions are about your **UTI symptoms and pain or discomfort in your lower abdomen, genitals and/or bladder** related to your UTI(s). Please indicate whether you have experienced any of the following symptoms **related to UTI** in the PAST 24 HOURS, and if so, how SEVERE they were:
C1	Needing to urinate more frequently than normal.
C2	Needing to urinate more urgently or more suddenly than normal.
C3	Feeling as though you have the urge to urinate despite having just urinated.
C4	Urine with an unusually strong or unpleasant smell.
C5	Cloudy urine.
C6	Debris or floating particles in your urine.
C7	Pain or burning sensation when you are urinating.
C8	Pain or burning sensation within the 30 minutes after urinating.
C9	Pain or discomfort in your lower back.
C10	Pain or discomfort in your side/flank.
C11	Pain or discomfort radiating down into your legs.

*Note*: The full Recurrent Urinary Tract Infection Symptom Scale (RUTISS) and scoring instructions are available from the corresponding author, Dr Katherine A. Finlay.

#### 2.3.5 Reliability

The internal consistency of the final RUTISS was explored by computing Cronbach's alpha (α) coefficients for the general ‘rUTI severity’ factor and for each sub‐factor. Acceptable internal consistency was indicated by α > 0.70.^31^

#### 2.3.6 Readability

The required literacy level for comprehension of the final RUTISS was estimated using the Automated Readability Index (ARI), a readability measure applicable to non‐narrative text such as questionnaires.^32^

## 3 RESULTS

### 3.1 Preliminary bifactor model

The preliminary bifactor model consisting of one general factor (‘rUTI symptom and pain severity’: all 17 items) and four orthogonal sub‐factors (‘urinary symptoms’, items C1–C4; ‘urinary presentation’, items C5–C7; ‘UTI pain and discomfort’, items D1–D6; and ‘bodily sensations’, items D7–D10) successfully converged with MHRM estimation.

### 3.2 Preliminary model assumption checks

The model intercept parameters successively decreased as expected (see Table S2), demonstrating the absence of any disordered thresholds and consistent use of the 11‐point Likert scale. The assumption of monotonicity was therefore satisfied. Likelihood ratio Chi‐square analysis indicated the absence of any DIF based on age, biological sex, household income, education level or current antibiotic use (χ^2^, *p* > 0.05; see Table S3). No statistically significant group differences were found, demonstrating item invariance based on all five characteristics.

Examination of the model's residuals indicated local independence for 89.3% of item pairs (*Q*_3_ < 0.30). The mean residual correlation (*Q*_3_) for all item pairs was negligible (*M* = 0.07, *SD* = 0.13). However, there were eight cases of LID between the following item pairs: C1–C2, C3–C4, D1‐D4, D2–D3, D2–D6, D2–D7, D2–D7 and D5–D6 (*Q*_3_ > 0.30), suggesting possible redundance or the existence of another shared dimension. The preliminary bifactor model therefore did not fully meet the assumption of local independence, highlighting items for potential deletion (see Section 3.3).

#### 3.2.1 Preliminary model fit and performance

Collectively, the general ‘rUTI symptom and pain severity’ factor and the four orthogonal sub‐factors accounted for 79.6% of the common variance (see Table S2). The general factor individually accounted for 48.6%, and the sub‐factors accounted for between 5.7% and 9.2% each. All items loaded onto the general factor with standardised factor loadings above 0.30 (range = 0.49 – 0.95; see Table S2 for all factor loadings). Additionally, all items within the ‘urinary symptoms’ and ‘urinary presentation’ sub‐factors obtained standardised loadings greater than 0.30 (range = 0.46 – 0.78). However, items D2 (average pain when not urinating) and D3 (current pain) in the ‘UTI pain and discomfort’ sub‐factor and item D7 (bladder pain and pressure) in the ‘bodily sensations’ sub‐factor did not obtain standardised sub‐factor loadings greater than the expected minimum of 0.30 (see Table S2). All items indicated communalities greater than 0.60 as expected, except item C3 (perceived difficulty emptying the bladder) in the ‘urinary symptoms’ sub‐factor (*h* ^2^ = 0.53). Item fit statistics indicated good item fit for all items with item MNSQ outfit between 0.50 and 2.00 except items D2 (MNSQ = 3.44), D3 (MNSQ = 6.62) and D7 (MNSQ = 2.01; see Table S2 for all item fit statistics).

Evaluation of the slope parameters (α) for each item demonstrated that all items had at least ‘moderate’ discrimination capability (α > 0.64), with 12 out of 17 items (70.6%) demonstrating ‘high’ or ‘very high’ discrimination on the general factor and/or the relevant sub‐factor (α > 1.35).^28^ Items D2 and D3 showed very high discrimination capability within the general factor (α > 1.70).^28^ However, given low capability within the ‘UTI pain and discomfort’ sub‐factor (α = 0.04 and α = 0.12, respectively), both items were considered for deletion (see Section 3.3).^28^

The C_2_ goodness of fit test for ordinal data yielded a statistically significant result, suggesting poor overall model fit: C_2_ (102, *N* = 389) = 422.9, *p* < 0.05.^29^ Whereas the CFI suggested good fit (CFI = 0.97), the RMSEA and SRMSR indices both indicated inadequate fit (RMSEA = 0.099, 95% CI [0.090, 0.110]; SRMSR = 0.068).

#### 3.2.2 Preliminary model refinement

Applying the refinement strategy outlined in Section 2.3, poor fitting items were deleted one at a time, re‐running the model analysis iteratively after each proposed deletion to examine the consequences for both item and model fit. In total, six items were deleted, comprising as follows: item C3 (perceived difficulty emptying the bladder) due to a low communality (*h* ^2^ < 0.60) and contributing to an item pair with LID (*Q*_3_ > 0.30); item D1 (average pain when urinating) due to contributing to LID; item D2 (average pain when not urinating) due to demonstrating a low standardised factor loading (< 0.30), poor item MNSQ fit (>2.00), low discrimination capability (α < 0.65) and contributing to LID; item D3 (current pain) due to demonstrating a low standardised factor loading, poor item MNSQ fit, low discrimination capability and contributing to LID; item D6 (urethral pain when not urinating) due to contributing to LID; and item D7 (bladder pain and pressure) due to demonstrating a low standardised factor loading, poor item MNSQ fit and contributing to LID.

### 3.3 Final bifactor model

**FIGURE 2:**
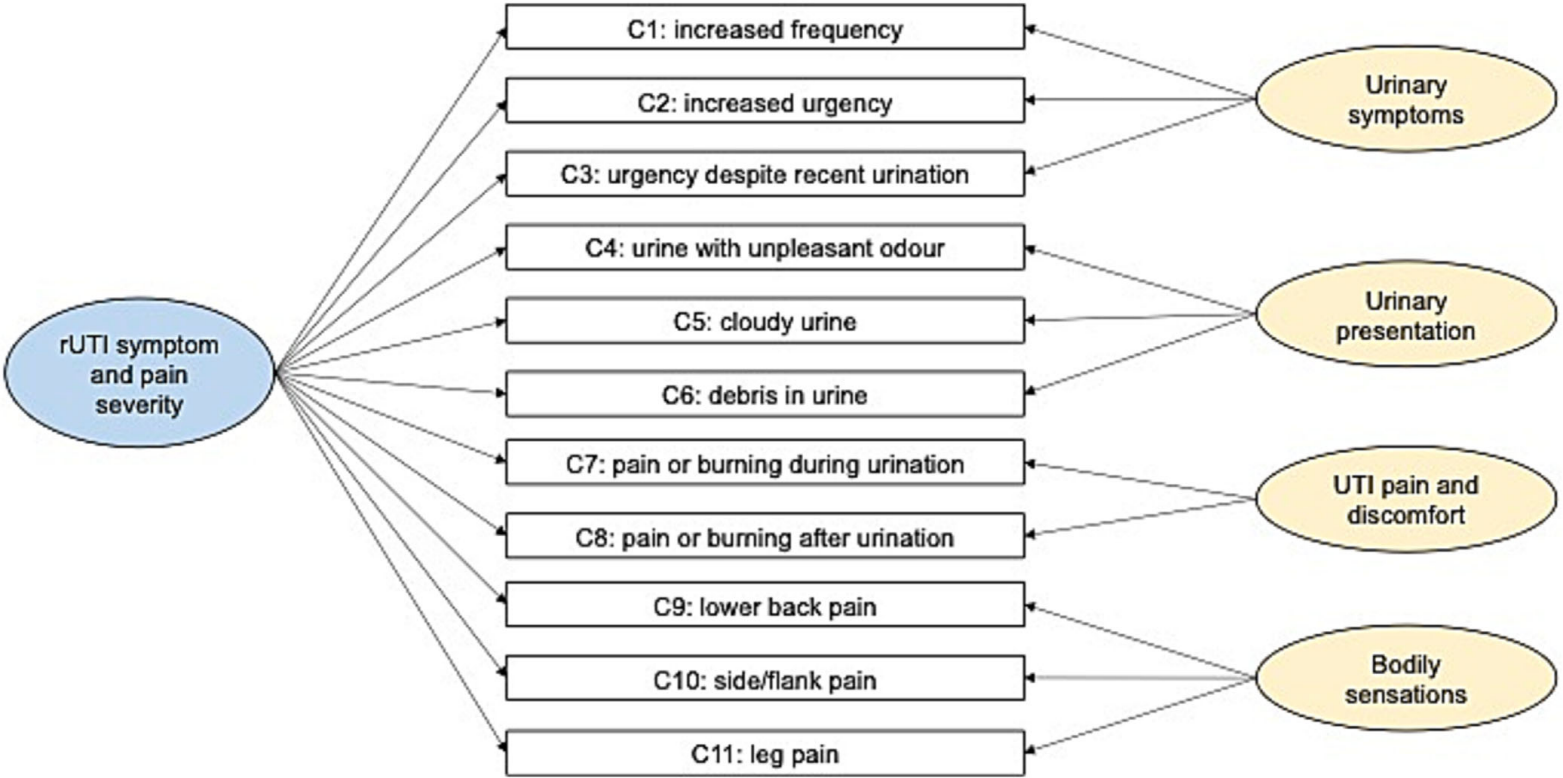
This diagram illustrates the bifactor structure represented by the 11 symptom and pain severity items included in the Recurrent Urinary Tract Infection Symptom Scale (RUTISS). All 11 items load onto the general factor coloured in blue on the left‐hand side: ‘rUTI symptom and pain severity’. Each item also loads onto a sub‐factor (coloured in yellow on the right‐hand side), measuring a more specific rUTI symptom trait. Standardised factor loadings (> 0.30), communalities (> 0.60) and model fit indices (root mean square error of approximation [RMSEA] = 0.041, comparative fit index [CFI] = 0.995, standardised root mean square residual [SRMSR] = 0.047) indicated excellent fit and structural validity. See Table 3 for the bifactor model parameters and factor loadings.

Removing items C3, D1, D2, D3, D6 and D7 resulted in a final 11‐item confirmatory bifactor model (see Figure 2), comprising one general factor (‘rUTI symptom and pain severity’) and four orthogonal sub‐factors (‘urinary symptoms’, items C1, C2, C4; ‘urinary presentation’, items C5, C6, C7; ‘UTI pain and discomfort’, items D4, D5; and ‘bodily sensations’, items D8, D9, D10). The final version of the RUTISS was created based upon this confirmatory bifactor model with updated item numbering, overall comprising 15 items: the 3‐item symptom frequency section, the 1‐item GRC scale and the 11‐item rUTI symptom and pain severity scale (see Table 2 for final included items; the full RUTISS and scoring instructions are available from the corresponding author).

#### 3.3.1 Final model assumption checks

The final model's intercept parameters successively decreased as expected (see Table 3), demonstrating no disordered thresholds and meeting the assumption of monotonicity. Likelihood ratio Chi‐square analysis indicated no statistically significant group differences in model functioning based on age, biological sex, household income, education level or current antibiotic use (χ^2^, *p* > 0.05; see Table S4). Therefore, no DIF was found, and the assumption of item invariance was met. Finally, no cases of LID were identified (*Q*_3_ < 0.30; *M* = 0.01, *SD* = 0.06), meeting the assumption of local independence.

**TABLE 3:** Bifactor graded response model item parameter estimates, fit statistics and factor structure.

Item	Slope					Intercept										Standardised factor loading					*h* ^2^	Item MNSQ outfit
	α^G^	α^S1^	α^S2^	α^S3^	α^S4^	c_1_	c_2_	c_3_	c_4_	c_5_	c_6_	c_7_	c_8_	c_9_	c_10_	G	S1	S2	S3	S4		
C1	5.54	3.85				5.29	4.04	2.42	1.05	−0.27	−2.21	−3.65	−6.43	−9.40	−11.0	0.80	0.55				0.94	0.87
C2	4.19	2.42				3.35	2.30	1.16	0.11	−0.57	−1.84	−2.80	−4.50	−6.75	−8.04	0.82	0.47				0.89	0.97
C4	2.75	0.85				1.90	1.23	0.55	−0.02	−0.49	−1.14	−1.73	−2.67	−3.59	−4.58	0.82	0.31				0.74	1.29
C5	2.49		1.98			0.26	−0.60	−1.20	−1.63	−1.97	−2.60	−3.26	−4.08	−5.25	−6.04	0.69		0.55			0.78	0.99
C6	3.95		3.65			1.33	0.02	−1.11	−1.81	−2.77	−4.11	−4.86	−6.28	−8.15	−9.25	0.70		0.65			0.91	0.90
C7	1.80		1.84			0.35	−0.42	−1.00	−1.47	−1.70	−2.46	−2.82	−3.47	−4.21	−4.87	0.58		0.60			0.70	0.77
D4	3.74			2.38		1.58	0.02	−0.86	−1.71	−2.25	−2.89	−3.58	−4.71	−6.28	−8.09	0.79			0.50		0.87	0.76
D5	3.14			2.44		1.42	0.29	−0.26	−0.97	−1.84	−2.77	−3.58	−4.52	−6.03	−7.45	0.73			0.56		0.85	0.64
D8	2.18				2.80	0.17	−1.25	−1.83	−2.26	−2.80	−3.70	−4.25	−5.23	−6.19	−7.62	0.55				0.71	0.81	0.85
D9	2.62				3.04	−0.76	−1.99	−2.74	−3.18	−2.93	−4.92	−5.44	−6.72	−7.25	−8.76	0.60				0.70	0.85	0.74
D10	1.35				1.89	−1.44	−2.27	−2.73	−3.03	−3.28	−3.65	−4.09	−4.97	−7.11	−8.27	0.47				0.66	0.65	0.89
ECV																0.48	0.05	0.10	0.05	0.13		

*Note*: G = general factor (rUTI symptom and pain severity); S1 = sub‐factor 1 (urinary symptoms); S2 = sub‐factor 2 (urinary presentation); S3 = sub‐factor 3 (UTI pain and discomfort); S4 = sub‐factor 4 (bodily sensations). *h* ^2^ = communality. α = slope (or discrimination) parameters; higher slopes indicate greater discrimination. ‘moderate’ discrimination capability: α = 0.65–1.34; ‘high’ discrimination capability: α = 1.35–1.69; ‘very high’ discrimination capability: α ≥ 1.70.^28^ c_1_–c_10_ = intercept parameters; these should successively decrease in value between c_1_ and c_10_ to demonstrate monotonicity.^12^ Intercept parameters are inversely proportional to threshold parameters (β), which are expected to successively increase alongside the response categories to satisfy the monotonicity assumption.^12^ Item MNSQ fit statistics between 0.50 and 2.00 are interpreted as acceptable for measurement, with statistics closer to 1.0 indicating best fit to the model with the least distortion.^27^

Abbreviations: ECV, explained common variance; MNSQ, mean square (fit statistics).

#### 3.3.2 Final model fit and performance

Collectively, the general ‘rUTI symptom and pain severity’ factor and the four orthogonal sub‐factors accounted for 81.7% of the common variance (see Table 3). The general factor individually accounted for 48.4% of the common variance, whereas the sub‐factors accounted for between 5.2% and 12.9% each. All items loaded onto the general factor and relevant sub‐factor with standardised factor loadings above 0.30 and communalities greater than 0.60 (see Table 3). All items demonstrated strong fit to the model, with MNSQ outfit statistics falling between the expected range of 0.50 – 2.00 (0.64 – 1.29, *M* = 0.88, *SD* = 0.17; see Table 3). Evaluation of the slope parameters demonstrated that all items possess at least moderate discrimination capability within the general factor and the relevant sub‐factor (α > 0.64), with 10 out of 11 items (90.9%) demonstrating ‘high’ or ‘very high’ discrimination capability (α > 1.34; see Table 3).

The model RMSEA, SRMSR and CFI demonstrate excellent model fit with values below the specified cut‐off values for IRT modelling: RMSEA = 0.041, CFI = 0.995 and SRMSR = 0.047.^29^, ^30^ The C_2_ goodness of fit test yielded a statistically significant result: C_2_ (33, *N* = 389) = 54.6, *p* < 0.05. Due to the test's sensitivity to sample size, model fit inferences were based on the RMSEA, SRMSR and CFI.^29^, ^30^

#### 3.3.3 Reliability

The internal consistency of the 11‐item general factor, ‘rUTI symptom and pain severity’, was excellent: Cronbach's α = 0.90.^31^ The internal consistency of the four sub‐factors was good: Cronbach's α = 0.89 for ‘urinary symptoms’, Cronbach's α = 0.86 for ‘urinary presentation’, Cronbach's α = 0.87 for ‘UTI pain and discomfort’ and Cronbach's α = 0.82 for ‘bodily sensations’.^31^

#### 3.3.4 Readability

The ARI for the final RUTISS is 7.0, indicating that this PROM is suitable for people with a reader's age of 12 years old and above (7th US grade, equivalent to UK Key Stage 3/Year 8).^32^

### 3.4 RUTISS scoring

Given the psychometrically confirmed bifactor structure of the RUTISS, questionnaire administrators may compute an overall RUTISS severity score that aligns with the general factor (‘rUTI symptom and pain severity’) and four individual domain scores for specific rUTI traits that align with the four orthogonal sub‐factors. Detailed within the scoring instructions that accompany the RUTISS (available from the corresponding author), individual domain scores may be calculated by summing the item scores relevant to the domain in question. The ‘urinary symptoms’, ‘urinary presentation’ and ‘bodily sensations’ domain therefore have a maximum possible score range of 0 – 30, and the ‘UTI pain and discomfort’ domain has a maximum possible score range of 0 – 20. For the overall RUTISS severity score, a scaled score with a maximum range of 0 – 100 is recommended to facilitate interpretation and comparison. This can be calculated by summing the four individual domain scores, dividing this figure by 11 (the number of items completed) and multiplying this by 10.

#### 3.4.1 Observed RUTISS scores

In this sample (*N* = 389), the average overall RUTISS severity score was 29.7 based on the final questionnaire items (*SD* = 22.1, range = 0 – 98.2). The average individual domain scores were *M* = 12.5 for ‘urinary symptoms’ (*SD* = 9.01, range = 0 – 30), *M* = 8.36 for ‘urinary presentation’ (*SD* = 8.63, range = 0 – 30), *M* = 6.35 for ‘UTI pain and discomfort’ (*SD* = 6.23, range = 0 – 20) and *M* = 5.41 for ‘bodily sensations’ (*SD* = 7.11, range = 0 – 28). The sample's scores therefore demonstrated heterogeneity in symptom severity, obtaining scores across the full breadth of possible scores. Clinical validation of the RUTISS to determine the questionnaire's sensitivity to change (responsiveness) and scoring interpretation information is ongoing.

Linear regression analyses confirmed previous findings that the GRC scale statistically significantly negatively predicts rUTI symptom and pain severity across all individual domain scores and the overall RUTISS severity score (*p* < 0.001; see Table S5), indicating that patient perceived improvement in symptoms predicts lower severity scores.^9^

## 4 DISCUSSION

The RUTISS is a patient‐generated and patient‐tested questionnaire evaluating the patient‐reported experience of rUTI symptoms.^9^ Developed in accordance with best practice recommendations with international patient and clinical input,^10^, ^11^ initial pilot testing indicated excellent psychometric properties.^9^ Exploratory analysis demonstrated strong content validity, test–retest reliability, internal consistency, construct validity and structural validity.^9^ The current study employed modern confirmatory validation approaches in order to refine the RUTISS and confirm the questionnaire's factor structure, as is the next required stage of PROM development.^10^ An international, clinically diverse sample reporting rUTI symptom experiences across the full spectrum of RUTISS scores was achieved.

The final 15‐item questionnaire demonstrates a clear, well‐fitted bifactor structure that minimises respondent burden whilst optimally maintaining the breadth of rUTI symptom challenges experienced by this vast patient cohort.^4^, ^12^, ^15^ Three items evaluate frequency of UTI symptoms, one item assesses patient‐perceived change in symptoms (GRC scale) and a further 11 items constitute the bifactor model's general factor measuring ‘rUTI symptom and pain severity’. Four sub‐factors are indicated by these 11 items: ‘urinary symptoms’ (3 items), ‘urinary presentation’ (3 items), ‘UTI pain and discomfort’ (2 items) and ‘bodily sensations’ (3 items). Scoring and administration instructions are supplied with the questionnaire, available from the corresponding author. It is recommended clinicians and researchers consider critical clinical features in conjunction with RUTISS scores (e.g., diabetes, menopause status, pregnancy and urinary catheterisation). An rUTI‐specific PROM assessing QoL impact, such as the Recurrent UTI Impact Questionnaire (RUTIIQ),^33^ should also be administered.

Items included in the final RUTISS loaded highly onto specific sub‐factors as well as the general factor; thus, the four specific rUTI symptom traits can be separated from the general, overarching symptom factor.^13^ Both bifactor model and item fit statistics were excellent,^29^, ^30^ demonstrating the strength of the questionnaire and that both an overall RUTISS severity score may be computed as well as individual domain scores. Internal consistency and reliability of the general factor and sub‐factor scales was high, meeting gold‐standard recommendations.^11^ It was observed that items assessing urinary frequency and urgency (C1 and C2) as well as pain and discomfort during and after urination (C7 and C8) contributed most to the general ‘rUTI symptom and pain severity’ factor.

Further research would work to address certain limitations. Whilst rUTI is significantly more common in females,^2^ additional testing of the RUTISS would be beneficial to assess its psychometric properties in males or people identifying as non‐binary. It is also acknowledged that most participants in this study were Caucasian, resided in high‐income countries and reported a high level of education and household income. Further research is necessary to establish cross‐validation of this questionnaire and develop translations for non‐English speaking populations or lower socioeconomic status respondents. Additionally, future research establishing regional differences in clinical practices of UTI management would be beneficial. Whilst the sample size was adequate, conducting GRM IRT analyses with a larger sample may further improve the reliability of model parameter estimates.^16^ Research to determine the responsiveness of the RUTISS to clinical changes (including urine culture) and to guide clinical interpretation of scoring is ongoing, as the next required stage in PROM development.^10^ Similarly, investigation of symptom variability using a repeated‐measures design monitored by the RUTISS would further provide insight into timeline progression and presentation of rUTI.

### 4.1 Conclusion

The RUTISS is a patient‐generated, psychometrically robust questionnaire comprising 15 items, assessing symptom frequency (3 items), patient‐perceived change in symptoms (1 item), and rUTI symptom and pain severity (11 items). This new PROM offers clinicians and researchers the unique opportunity to supplement established clinical testing methods with the patient perspective. Simple scoring allows for straightforward assessment and standardised monitoring of patient symptoms as well as patient‐perceived improvement or worsening in response to medical and non‐medical interventions.

## AUTHOR CONTRIBUTIONS

All authors contributed to the study conceptualisation and methodology. Material preparation and data collection were conducted by Abigail F. Newlands. Melissa Kramer and Jessica L. Price contributed to participant recruitment via Live UTI Free. Data analysis was conducted by Abigail F. Newlands and Katherine A. Finlay. The original draft of the manuscript was written by Abigail F. Newlands, and all authors commented on previous versions of the manuscript. All authors read and approved the final manuscript.

## CONFLICT OF INTEREST STATEMENT

Melissa Kramer is CEO of Live UTI Free Ltd.; however, no financial incentives have been received.

## Supporting information


**Figure S1.** Sampling flow chart.

**Table S2.** Preliminary bifactor graded response model item parameter estimates, fit statistics, and factor structure.

**Table S3.** Differential item functioning analysis results of the preliminary RUTISS model.

**Table S5.** Linear regression results predicting RUTISS severity scores from global rating of change scale.

**Table S1.** Statistical terminology definitions.

**Table S4.** Differential item functioning analysis results of the final RUTISS.
