# Supplementary figures and images for: Confirmatory structural validation and refinement of the Recurrent Urinary Tract Infection Symptom Scale
Source: BJUI Compass. 2023 Oct 4;5(2):240–52. doi: 10.1002/bco2.297 (PMC10869661; doi:10.1002/bco2.297)

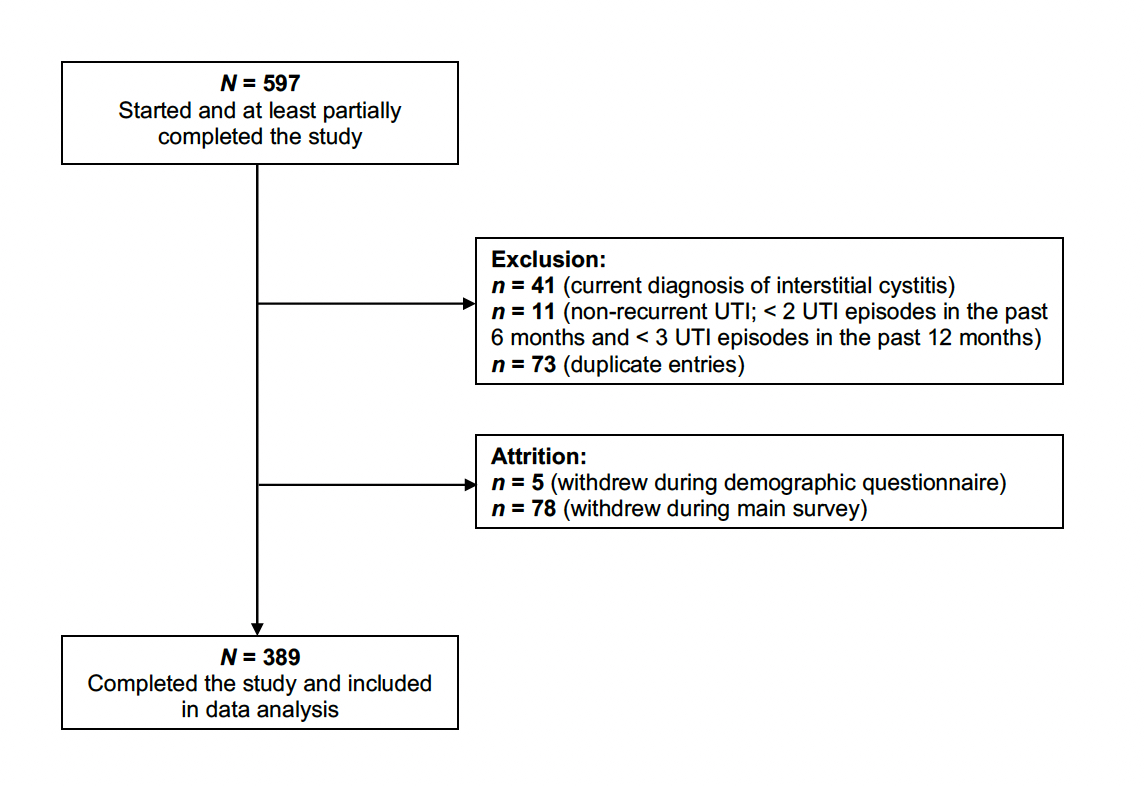

Supplement: Supplementary file 1 — Figure S1. Sampling flow chart. [file BCO2-5-240-s003.jpg]
