# Supplementary material for: Confirmatory structural validation and refinement of the Recurrent Urinary Tract Infection Symptom Scale
Source: BJUI Compass. 2023 Oct 4;5(2):240–52. doi: 10.1002/bco2.297 (PMC10869661; doi:10.1002/bco2.297)
Supplement: Supplementary file 2 — Table S2. Preliminary bifactor graded response model item parameter estimates, fit statistics, and factor structure. [file BCO2-5-240-s006.docx]

**Table S2.** Preliminary bifactor graded response model item parameter estimates, fit statistics, and factor structure

| Item | Slope | | | | | Intercept | | | | | | | | | | Standardised factor loading | | | | | *h*^2^ | Item MNSQ outfit |
| --- | --- | --- | --- | --- | --- | --- | --- | --- | --- | --- | --- | --- | --- | --- | --- | --- | --- | --- | --- | --- | --- | --- |
|  | α^G^ | α^S1^ | α^S2^ | α^S3^ | α^S4^ | c_1_ | c_2_ | c_3_ | c_4_ | c_5_ | c_6_ | c_7_ | c_8_ | c_9_ | c_10_ | G | S1 | S2 | S3 | S4 |  |  |
| C1 | 3.81 | 3.92 |  |  |  | 4.40 | 3.36 | 2.00 | .86 | –.21 | –1.79 | –2.95 | –5.22 | –7.69 | –9.01 | .67 | .68 |  |  |  | .91 | 1.67 |
| C2 | 3.45 | 3.63 |  |  |  | 3.50 | 2.41 | 1.22 | .14 | –.55 | –1.88 | –2.87 | –4.60 | –6.94 | –8.29 | .65 | .69 |  |  |  | .90 | 1.70 |
| C3 | 1.39 | 1.13 |  |  |  | 1.24 | .52 | –.07 | –.37 | –.61 | –.96 | –1.36 | –2.10 | –3.02 | –3.91 | .56 | .46 |  |  |  | **.53** | 1.18 |
| C4 | 2.31 | 1.71 |  |  |  | 1.91 | 1.25 | .59 | .02 | –.45 | –1.09 | –1.70 | –2.63 | –3.57 | –4.59 | .69 | .51 |  |  |  | .74 | 1.68 |
| C5 | 1.89 |  | 2.51 |  |  | .32 | –.55 | –1.15 | –1.59 | –1.92 | –2.55 | –3.22 | –4.05 | –5.22 | –5.99 | .53 |  | .70 |  |  | .77 | 1.41 |
| C6 | 2.88 |  | 4.20 |  |  | 1.37 | .09 | –1.00 | –1.68 | –2.61 | –3.90 | –4.63 | –5.99 | –7.79 | –8.85 | .54 |  | .78 |  |  | .90 | 1.15 |
| C7 | 1.5D0 |  | 2.07 |  |  | .38 | –.39 | –.98 | –1.44 | –1.67 | –2.43 | –2.79 | –3.44 | –3.18 | –4.85 | .49 |  | .67 |  |  | .69 | .96 |
| D1 | 3.06 |  |  | 1.71 |  | 2.48 | 1.02 | .12 | –.92 | –1.65 | –2.48 | –3.03 | –4.56 | –6.48 | –8.10 | .79 |  |  | .44 |  | .81 | 1.13 |
| D2 | 5.31 |  |  | **.04** |  | 5.04 | 2.62 | 1.5 | –.33 | –1.31 | –2.60 | –4.07 | –6.25 | –8.63 | –10.7 | .95 |  |  | **.01** |  | .91 | **3.44** |
| D3 | 4.83 |  |  | **.12** |  | 3.96 | 1.88 | .34 | –1.00 | –2.30 | –3.52 | –4.79 | –6.57 | –9.01 | –11.2 | .94 |  |  | **.02** |  | .89 | **6.62** |
| D4 | 4.35 |  |  | 4.21 |  | 2.26 | .19 | –1.03 | –2.24 | –3.00 | –3.91 | –4.89 | –6.44 | –8.42 | –10.6 | .69 |  |  | .67 |  | .93 | .97 |
| D5 | 2.89 |  |  | 1.84 |  | 1.39 | .36 | –.17 | –.82 | –1.61 | –2.45 | –3.17 | –4.00 | –5.36 | –6.62 | .76 |  |  | .48 |  | .80 | .95 |
| D6 | 2.31 |  |  | .97 |  | 1.59 | .75 | .13 | –.54 | –1.11 | –1.70 | –2.14 | –2.88 | –4.05 | –4.95 | .76 |  |  | .32 |  | .68 | 1.40 |
| D7 | 3.02 |  |  |  | .72 | 2.46 | 1.35 | .69 | –.02 | –.79 | –1.64 | –2.31 | –3.47 | –4.48 | –5.74 | .85 |  |  |  | **.20** | .77 | **2.01** |
| D8 | 2.72 |  |  |  | 2.86 | .13 | –1.38 | –2.00 | –2.46 | –3.04 | –4.01 | –4.68 | –5.79 | –6.85 | –8.44 | .63 |  |  |  | .67 | .84 | .94 |
| D9 | 2.35 |  |  |  | 2.78 | –.74 | –1.86 | –2.54 | –2.95 | –3.65 | –4.59 | –5.09 | –6.31 | –6.81 | –8.22 | .59 |  |  |  | .69 | .82 | .73 |
| D10 | 1.47 |  |  |  | 1.69 | –1.44 | –2.25 | –2.70 | –2.99 | –3.24 | –3.60 | –4.03 | –4.91 | –7.03 | –8.18 | .52 |  |  |  | .60 | .63 | .76 |
| ECV |  |  |  |  |  |  |  |  |  |  |  |  |  |  |  | .49 | .08 | .09 | .06 | .08 |  |  |

*Note.* G = general factor (rUTI symptom and pain severity); S1 = sub-factor 1 (urinary symptoms); S2 = sub-factor 2 (urinary presentation); S3 = sub-factor 3 (UTI pain and discomfort); S4 = sub-factor 4 (bodily sensations). *h*^2^ = communality. MNSQ = mean square (fit statistics). ECV = explained common variance.

α = slope (or discrimination) parameters; higher slopes indicate greater discrimination. ‘moderate’ discrimination capability: α = .65 – 1.34; ‘high’ discrimination capability: α = 1.35 – 1.69; ‘very high’ discrimination capability: α ≥ 1.70 (28).

c_1_–c_10_ = intercept parameters; these should successively decrease in value between c_1_ and c_10_ to demonstrate monotonicity (12). Intercept parameters are inversely proportional to threshold parameters (β), which are expected to successively increase alongside the response categories to satisfy the monotonicity assumption (12).

Item MNSQ fit statistics between .50 and 2.00 are interpreted as acceptable for measurement, with statistics closer to 1.0 indicating best fit to the model with the least distortion (27).

Poor fitting items are identified with the following statistics in bold: Slope parameters (α) < .65; standardised factor loadings < .30; communalities (*h*^2^) < .60; item MNSQ fit statistics < .50 or > 2.00.
