# Supplementary material for: Confirmatory structural validation and refinement of the Recurrent Urinary Tract Infection Symptom Scale
Source: BJUI Compass. 2023 Oct 4;5(2):240–52. doi: 10.1002/bco2.297 (PMC10869661; doi:10.1002/bco2.297)
Supplement: Supplementary file 3 — Table S3. Differential item functioning analysis results of the preliminary RUTISS model. [file BCO2-5-240-s004.docx]

**Table S3.** Differential item functioning analysis results of the preliminary RUTISS model

| Item | Age | | | Biological sex | | | Household income | | | Education | | | Current antibiotic use | | |
| --- | --- | --- | --- | --- | --- | --- | --- | --- | --- | --- | --- | --- | --- | --- | --- |
|  | *χ*^2^ | *df* | *p* | *χ*^2^ | *df* | *p* | *χ*^2^ | *df* | *p* | *χ*^2^ | *df* | *p* | *χ*^2^ | *df* | *p* |
| C1 | 5.96 | 15 | 1.00 | 1.27 | 15 | 1.00 | 14.0 | 15 | 1.00 | 21.2 | 15 | 1.00 | 10.8 | 15 | 1.00 |
| C2 | 9.67 | 15 | 1.00 | 3.09 | 15 | 1.00 | 18.2 | 15 | 1.00 | 21.0 | 15 | 1.00 | 8.65 | 15 | 1.00 |
| C3 | 10.5 | 15 | 1.00 | .45 | 15 | 1.00 | 32.1 | 15 | .11 | 10.8 | 15 | 1.00 | 20.3 | 15 | 1.00 |
| C4 | 6.97 | 15 | 1.00 | 1.59 | 15 | 1.00 | 21.3 | 15 | 1.00 | 16.3 | 15 | 1.00 | 12.0 | 15 | 1.00 |
| C5* | 0.00 | 5 | 1.00 | 0.00 | 5 | 1.00 | 0.00 | 5 | 1.00 | 0.00 | 5 | 1.00 | 0.00 | 5 | 1.00 |
| C6* | 0.00 | 5 | 1.00 | 0.00 | 5 | 1.00 | 0.00 | 5 | 1.00 | 0.00 | 5 | 1.00 | 0.00 | 5 | 1.00 |
| C7 | 29.0 | 15 | .27 | 8.57 | 15 | 1.00 | 21.7 | 15 | 1.00 | 19.0 | 15 | 1.00 | 21.6 | 15 | 1.00 |
| D1 | 10.4 | 15 | 1.00 | .51 | 15 | 1.00 | 27.7 | 15 | .40 | 16.1 | 15 | 1.00 | 18.2 | 15 | 1.00 |
| D2 | 8.53 | 15 | 1.00 | 2.04 | 15 | 1.00 | 24.5 | 15 | .98 | 12.9 | 15 | 1.00 | 29.0 | 15 | .24 |
| D3 | 10.2 | 15 | 1.00 | 2.56 | 15 | 1.00 | 25.3 | 15 | .78 | 14.8 | 15 | 1.00 | 25.0 | 15 | .75 |
| D4 | 9.40 | 15 | 1.00 | 1.71 | 15 | 1.00 | 26.8 | 15 | .51 | 9.19 | 15 | 1.00 | 16.2 | 15 | 1.00 |
| D5 | 20.5 | 15 | 1.00 | 2.87 | 15 | 1.00 | 24.0 | 15 | 1.00 | 13.5 | 15 | 1.00 | 14.0 | 15 | 1.00 |
| D6 | 19.7 | 15 | 1.00 | 3.07 | 15 | 1.00 | 16.7 | 15 | 1.00 | 12.6 | 15 | 1.00 | 12.5 | 15 | 1.00 |
| D7 | 5.70 | 15 | 1.00 | .57 | 15 | 1.00 | 21.0 | 15 | 1.00 | 18.5 | 15 | 1.00 | 22.1 | 15 | 1.00 |
| D8 | 6.00 | 15 | 1.00 | 3.70 | 15 | 1.00 | 28.1 | 15 | .36 | 26.6 | 15 | .55 | 15.2 | 15 | 1.00 |
| D9 | 12.4 | 15 | 1.00 | 3.72 | 15 | 1.00 | 25.5 | 15 | .74 | 15.6 | 15 | 1.00 | 14.7 | 15 | 1.00 |
| D10 | 13.4 | 15 | 1.00 | .68 | 15 | 1.00 | 17.0 | 15 | 1.00 | 16.4 | 15 | 1.00 | 15.4 | 15 | 1.00 |

*Note.* *Items C5 and C6 were used as ‘anchor’ variables in the likelihood ratio DIF analysis, after determining through linear regression analyses that neither of them are statistically significantly predicted by any of these five characteristics (*p* > .05) (24). Their *χ*^2^ values therefore equate to zero.

DIF is identified if the *χ*^2^ test is statistically significant at *p* < .05. Here, no DIF is identified, and therefore responses to items in the revised RUTISS do not depend on age, biological sex, household income, level of education, or current antibiotic use (24). *p*-values have been adjusted for multiple comparisons using the Bonferroni correction (25).
