# Supplementary material for: Confirmatory structural validation and refinement of the Recurrent Urinary Tract Infection Symptom Scale
Source: BJUI Compass. 2023 Oct 4;5(2):240–52. doi: 10.1002/bco2.297 (PMC10869661; doi:10.1002/bco2.297)
Supplement: Supplementary file 4 — Table S5. Linear regression results predicting RUTISS severity scores from global rating of change scale. [file BCO2-5-240-s001.docx]

**Table S5.** Linear regression results predicting RUTISS severity scores from global rating of change scale

|  | *R^2^_Adj_* | *F*(1, 387) | β | 95% CI | |
| --- | --- | --- | --- | --- | --- |
|  |  |  |  | LB | UB |
| Individual domain score |  |  |  |  |  |
| Urinary symptoms | .11* | 46.7 | –1.21 | –1.55 | –.86 |
| Urinary presentation | .05* | 20.4 | –.79 | –1.13 | –.44 |
| UTI pain and discomfort | .14* | 62.8 | –.95 | –1.12 | –.71 |
| Bodily sensations | .07* | 29.0 | –.77 | –1.05 | –.49 |
| Overall RUTISS severity score | .14* | 63.1 | –3.37 | –4.21 | –2.54 |

*Note.* *N* = 389. CI = confidence interval; LB = lower bound; UB = upper bound.

**p* < .001.
