# Supplementary material for: Confirmatory structural validation and refinement of the Recurrent Urinary Tract Infection Symptom Scale
Source: BJUI Compass. 2023 Oct 4;5(2):240–52. doi: 10.1002/bco2.297 (PMC10869661; doi:10.1002/bco2.297)
Supplement: Supplementary file 5 — Table S1. Statistical terminology definitions. [file BCO2-5-240-s005.docx]

**Table S1.** Statistical terminology definitions

| Terminology | Definition |
| --- | --- |
| Bifactor model | A bifactor model is a latent structure that hypothesises that (i) there is a general factor that explains the shared variance between all the items, and (ii) there are two or more specific, uncorrelated factors which each account for the unique influence of the specific construct over and above the general factor. |
| Bonferroni correction | A multiple-comparison adjustment made to *p*-values when multiple statistical tests are conducted simultaneously. This reduces the chance of a Type I error (also known as ‘false-positive’) occurring. |
| Classical test theory (CTT) | Traditional psychometric methods, in contrast to item response theory (IRT). For example, exploratory and confirmatory factor analysis. |
| Comparative Fit Index (CFI) | A model fit index. With a maximum possible value of 1.00, CFI greater than .95 indicates excellent fit. |
| Communality (*h*^2^) | The proportion of a given item’s variance which is explained by the model’s factors. |
| Differential item functioning (DIF) | The extent to which an item may measure different abilities for individuals from different sub-groups, e.g., related to sociodemographic characteristics (e.g., age group, biological sex, etc.). To achieve item invariance, an item should be interpreted in the same way across different sub-groups (and therefore no DIF should be found). |
| Factor loading | The extent to which an item is related to a given factor. Loadings may be interpreted similarly to correlation coefficients. |
| Global rating of change (GRC) scale | A GRC scale measures self-perceived change in, e.g., health status over a given period. The GRC scale included in the RUTISS assesses change (worsening or improvement) over the past 24 hours. |
| Graded response model (GRM) | A mathematical model applied for grading/ordinal responses with polytomous response options. |
| Intercept parameter (c) | The intercept parameter is an bifactor IRT model parameter which governs the choice of the next category over the previous one (i.e., responding 10 versus 9). Intercept parameters are inversely proportional to threshold parameters (β), which are expected to successively increase alongside the response categories to satisfy the monotonicity assumption. |
| Internal consistency | A reliability index determining the extent to which the items included in a scale are correlated and thus measure the same construct and result in similar scores. Typically indicated by Cronbach’s α. |
| Item | A question in a scale or questionnaire. |
| Item response theory (IRT) | Developed largely in response to the limitations of classical test theory (CTT), item response theory… |
| Local independence | The assumption of IRT analysis that there is local independence of items after controlling for the latent construct. In other words, after controlling for the factor influencing a respondent’s choice of response, there should be no statistically significant association between items. Item pairs that do not meet this assumption are said to exhibit local item dependence, or LID. |
| Mean square (MNSQ) outfit | MNSQ fit statistics indicate the amount of distortion of a scale or measurement system. Outfit statistics are ‘outlier-sensitive’, meaning that they are more “sensitive to responses to items with difficulty far from a person, and vice-versa” (25). The expected value is 1.0, so values near 1.0 indicate little distortion. Values less than 1.0 may suggest that observations are too predictable (model overfit), and values greater than 1.0 may suggest that observations are too unpredictable (model underfit). Values falling between .50 and 2.00 indicating that they are acceptable for measurement. |
| Metropolis-Hastings Robbins-Monro (MHRM) estimation | The mathematical algorithm recommended to estimate multidimensional item response theory models with a high number of expected factors (>3). |
| Monotonicity | The assumption of IRT analysis that as the trait level (e.g., pain severity) increases, the probability of higher scores also increases. To meet the assumption and demonstrate consistent use of the 11-point scale, the intercept values were expected to successively decrease as the response categories, and therefore the latent trait of severity, increased. Intercept parameters are inversely proportional to threshold parameters (β), which are expected to successively increase alongside the response categories to satisfy the monotonicity assumption. |
| Multidimensionality | Multidimensional questionnaires measure more than one latent construct. In contrast to unidimensional questionnaires, in which only one factor accounts for an individual’s response to items. |
| Polytomous scale | An item with more than two response options uses a ‘polytomous’ scale (e.g., 5-point Likert scale). This contrasts with a dichotomous scale which employs only two response options (e.g., yes/no, agree/disagree, etc.). |
| Root mean square error of approximation (RMSEA) | A model fit index. With a minimum possible value of zero, RMSEA less than .06 indicates excellent fit. |
| Slope parameter (α) | Also called a discrimination parameter, the slope parameter is an IRT model parameter which indicates the strength of the relationship between an item and the measured construct (e.g., pain severity). The slope parameter also determines how well an item can discriminate or distinguish between respondents above and below a certain threshold. The larger the slope parameter, the more effective the item. |
| Standardised root mean square residual (SRMSR) | A model fit index. With a minimum possible value of zero, SRMSR less than .06 indicates excellent fit. |
| Structural validity | The extent to which scores of a scale adequately demonstrate the dimensionality or factor structure of the construct being measured (e.g., pain severity). |
| Theta (θ) | The level of the latent variable being measured by a scale (e.g., pain severity). |
| Yen’s *Q*_3_ | A residual correlation coefficient indicating the level of association between two items after controlling for the latent variable being measured. Typically interpreted as correlation coefficients, values greater than .30 may suggest local item dependence (LID), failing the IRT assumption of local independence. |

*Note.* For more detailed explanations and mathematical background, see reference numbers: 12, 13, 14, 15, 16, 20, 21, 22, 23, 24, 25, 26, 27, 28, 29.
